# Trajectory analysis in clinical research

**DOI:** 10.1093/ckj/sfag239

**Published:** 2026-08-04

**Authors:** Graziella D’Arrigo, Mercedes Gori, Nicholas C Chesnaye, Kitty J Jager, Friedo W Dekker, Merel van Diepen, Carmine Zoccali, Giovanni Tripepi

**Affiliations:** CNR-IFC, Institute of Clinical Physiology of Reggio Calabria, Italy; CNR-IFC, Institute of Clinical Physiology of Rome, Italy; ERA Registry, Amsterdam UMC location and the University of Amsterdam, Department of Medical Informatics, Amsterdam, The Netherlands; Amsterdam Public Health, Quality of Care, Amsterdam, The Netherlands; ERA Registry, Amsterdam UMC location and the University of Amsterdam, Department of Medical Informatics, Amsterdam, The Netherlands; Amsterdam Public Health, Quality of Care, Amsterdam, The Netherlands; Department of Clinical Epidemiology, Leiden University Medical Center, Leiden, The Netherlands; Department of Clinical Epidemiology, Leiden University Medical Center, Leiden, The Netherlands; Renal Research Institute, New York, NY, USA; Institute of Molecular Biology and Genetics (Biogem), Ariano Irpino, Italy; Associazione Ipertensione Nefrologia Trapianto Renale (IPNET), c/o Nefrologia, Grande Ospedale Metropolitano, Reggio Calabria, Italy; CNR-IFC, Institute of Clinical Physiology of Reggio Calabria, Italy; Fondazione Toscana “Gabriele Monasterio” of Pisa, Italy

**Keywords:** longitudinal data, STATA, trajectory analysis

## Abstract

Trajectory analysis investigates patterns of change over time of variables across various fields, including clinical and public health research. This method is useful for understanding the evolution of behaviors, conditions, and biomarkers (such as serum creatinine, blood pressure, etc.) over time. In medicine, by examining trajectories, researchers can identify distinct clinical patterns and assess the impact of risk factors and interventions on these patterns. This paper explores some theoretical and practical aspects of trajectories analysis by focusing on group-based trajectory models (GBTM) and emphasizing the importance of this approach in clinical research by providing guidance on its implementation using STATA software.

## INTRODUCTION

Trajectory analysis is an advanced statistical method used to examine how variables change over time, with the ultimate goal of identifying groups of individuals who share similar patterns of evolution. A major strength of trajectory analysis lies in its ability to capture the shape and potential non-linear nature of temporal changes. For this reason, the trajectory analysis is particularly valuable in clinical research, where understanding the non-linear progression of behaviors, clinical conditions, biomarkers, or health outcomes is essential for enhancing treatment and care strategies as well as to better assess the impact of risk factors on certain outcomes [1, 2] such as cardiovascular events, hospitalization, or mortality [3]. Additionally, trajectories can be included as covariates in multivariable models to assess their independent effects on outcomes, while adjusting for confounders [1, 4] (e.g. age, sex, treatment regimens, or baseline health status). Graphical representations of trajectory groups, supported by many statistical software packages, further enhance the clarity and communicability of findings for researchers, clinicians, and policymakers [5, 6].

D’Arrigo *et al*. [7] applied this method to investigate the relationship between repeated venous bicarbonate levels and a predefined composite renal outcome (≥30% estimated glomerular filtration rate reduction, dialysis, or transplantation) in patients with chronic kidney disease (CKD) stages 2–5. To this end, the authors sought to identify clusters of patients with similar trajectories of venous bicarbonate levels over time and distinguished four distinct patterns. Thus, patients were classified into four groups: consistently low, moderate, moderate–high, and high bicarbonate levels. In survival analyses, a significant inverse association was found between venous bicarbonate trajectories and the risk of renal outcomes, independent of all potential confounders, so that patients in the higher bicarbonate trajectory groups experienced a progressively lower risk of reaching the composite renal endpoint [7].

The versatility of trajectory analysis is supported by a range of statistical techniques, including growth curve modeling, latent class growth analysis (LCGA), group-based trajectory modeling (GBTM), and mixed-effects models. In this paper, we focus on GBTM [1, 2, 8] to illustrate the practical applications of trajectory analysis, emphasizing its relevance in clinical research. GBTM is a method used to examine how individuals’ health changes over time. Rather than assuming that everyone follows the same pattern, GBTM identifies subgroups of patients who share similar health trajectories. In nephrology research, e.g. GBTM can be applied to track changes in kidney function among patients with CKD. Instead of focusing only on the average rate of decline, GBTM uncovers distinct patterns—such as patients whose kidney function remains stable for many years, those who decline gradually, and others who experience rapid deterioration. By revealing these different pathways, GBTM helps researchers gain a deeper understanding of CKD progression. This, in turn, supports more personalized care and more informed decisions around treatment and monitoring. In this paper, we also provide practical guidance on conducting trajectory analysis using STATA [9].

## STEPS TO BE FOLLOWED TO PERFORM A GBTM

### Step 1–consider the nature and the distribution of the variables used to define the trajectories

Before starting the analysis, it is essential to consider the type of the variable used to define the trajectories (a continuous or rating scale, binary outcome, or a count) as well as, when the distribution is normal (or normalized), to identify the minimum and maximum value of the variable to be investigated (such information is required to run the STATA command). Technically speaking, for modeling a rating scale or continuous variable, the censored normal distribution is used by the GBTM model. When dealing with counts, one may use the (zero-inflated) Poisson model and for binary outcomes, the logistic regression approach [6, 8]. For a more detailed description of the analytical approaches for modeling continuous longitudinal data that are poorly fit by the normal distribution even with censoring, see [10].

### Step 2–define the number of distinct groups

When possible, based on previous articles, researchers should consider a number of groups identical to that observed in similar contexts and to assess how does this number fits the data. If this information about the number of groups is lacking, various numbers of groups can be considered. An empirical rule is that none of the resulting groups should include less than 5% of the total cases [2]. Overall, the identification of the number of groups and pattern (i.e. shape) of trajectories can be considered as a “trial and error iterative process” (see steps 3–4), where a researcher combining various criteria aims to identify the best fitting model.

### Step 3–modeling considerations

To determine the optimal shape of each trajectory, various functions such as linear, quadratic, and cubic polynomials can be investigated. Statistical criteria, such as *p*-values, are used to guide this process.

### Step 4–assess the model fit

In order to assess the goodness of fit (which pertains both the number of groups and the type of trajectories), it is needed to consider the following items:

- Bayesian information criteria (BIC). The model with the highest (least negative) value of BIC is the preferred one. BIC is preferred over Akaike Information Criterion (AIC) as it imposes a stronger penalty for model complexity, thereby reducing the likelihood of over-extracting trajectory groups.
- Statistical significance of regression coefficients.
- Group size (>5%).
- Graphical representations of trajectories by visually assessing the overlap between observed and estimated shapes.
- Average posterior probabilities (APPs) of assignments (APPs > 0.70). The APP measures how confidently patients are assigned to their respective trajectory groups. For each patient, the model calculates the probability of belonging to each group based on their observed data. The APP for a group is the average of these probabilities for all individuals classified into that group. A high APP (>0.70) indicates good classification accuracy, meaning individuals are clearly distinguished and well-matched to their assigned trajectory group. On the contrary, low APP suggests more uncertainty in group membership, which may undermine the reliability of the trajectory solution.
- Odds of correct classification (OCC > 5). OCC expresses how likely the model accurately classifies items in each group. OCC is based on the maximum likelihood classification method and the estimated size of each group.
- Entropy (>0.80). The entropy is a global measure of classification uncertainty and indicates that individuals are well classified and that there is adequate separation between groups [2].

If these conditions are not met, there are two options: (1) reassess and possibly reduce or increase the number of groups to improve model fit and classification quality; (2) consider alternative modeling approaches or re-examine the data for outliers or noise that may affect group identification.

Both statistical criteria and visual inspection are jointly considered within the same iterative model selection process. The order of the bullets is meant for clarity of presentation rather than to indicate a hierarchy among the criteria: all criteria, including visual inspection, are considered together during the iterative evaluation of competing trajectory models.

Overall, model selection follows an iterative approach based on BIC, parsimony, classification quality, and interpretability. The significance (*p*-values) of polynomial terms was used during model refinement to define trajectory shapes, but not as a primary model selection criterion.

## PRACTICAL EXAMPLE

To explain the procedure for the identification of trajectories, we consider a hypothetical cohort of 109 CKD patients who completed SF-36 questionnaires during a 3-year follow-up period. The aim of the study is to assess the evolution of the mental component score (MCS) as derived by the SF-36 (Short Form-36 Health Survey) questionnaire [11] over time. MCS was measured at baseline and at 12, 24, and 36 months. To address the research question, we used GBTM to assess how MCS changes over time and to identify homogeneous patterns (trajectories) of this change [12].

### Step 1–consider the nature and the distribution of the variable used to define the trajectories

For the scope of this paper, GBTM was applied using the “*traj*” command in STATA software [9]. As said, the first step in trajectory analyses is to examine the distribution of the outcome variable. In this case MCS, being a score of a rating scale with values ranging from 0 to 100 (the higher the score, the better the patient’s mental health), we opted for a censored normal distribution, which is well suited for modeling the distributions of psychometric scales by defining their minimum and maximum values. The trajectory analyses by STATA require adequately structured databases, and Table 1 shows how such databases must be structured. Each row corresponds to an individual. The outcome variable of interest (MCS) must be reported across different time points in separate columns (MCS0–MCS3), along with information about the days corresponding to the time spanning from baseline to each time point of data collection (*T*0–*T*3).

**Table 1:** Structure of the database for the first seven patients included in the study.

	Mental component score (MCS)				Time points (days)			
Patients’ ID	MCS_0_	MCS_1_	MCS_2_	MCS_3_	*T*_0_	*T*_1_	*T*_2_	*T*_3_
1	44	38	39	29	0	364	720	1056
2	39	42	46	46	0	366	727	1077
3	36	39	26	38	0	366	699	1091
4	52	49	39	55	0	371	735	1092
5	31	24	27	26	0	363	741	1106
6	43	47	45	52	0	364	727	1056
7	29	17	45	43	0	364	742	1134
…	…	…	…	…	…	…	…	…

If the time points at which the measurements are made importantly differ among patients, a bias is very likely. On the other hand, the user-written command we applied (traj – from Jones and Nagin’s method), expects balanced panel data or equally spaced time points. GBTM can also accommodate incomplete longitudinal data through maximum likelihood estimation using all available observations; however, non-random missingness may still bias parameter estimates and should therefore be carefully explored and assessed by expert statisticians.

### Step 2–define the number of distinct groups

For a given variable, trajectories may exhibit linear, quadratic, or cubic trends, depending on the number of time points available during follow-up. As a general rule, a linear trajectory (order = 1) can be tested to describe the evolution of a variable measured at two time points, a quadratic trajectory (order = 2) if the variable is measured at three time points, and a cubic trajectory (order = 3) if there are ≥4 time points. Thus, the order of the trajectory corresponds to “*n−1”*, where “*n*” represents the number of available time points.

If there is no previous information in literature about the number of trajectories to be tested, methodologists recommend to start by testing two trajectories. Following this pragmatic rule and having a database with four points in time, we begin with two cubic-order trajectories (as their order would be 3, these two trajectories are indicated as “3 3”).

### Step 3–modeling considerations

As reported in Table 2a, both cubic-order trajectories were not statistically significant (*p* = 0.91 and *p* = 0.94), indicating that they were inadequate to describe the MCS pattern over time. Thus, we lower the order to 2 and now test two quadratic-order trajectories (“2 2”) and look at the statistical significance of those two patterns. As reported in Table 2b, also both quadratic-order trajectories failed to be statistically significant (*p* = 0.07 and *p* = 0.58), and for this reason, also they are not adequate to describe the MCS evolution across time. As the coefficients in both models featuring two trajectories are not statistically significant, we continue with the use of a model incorporating three trajectories for both cubic and quadratic orders (Table 3).

**Table 2:** Stata outputs of model with 2 trajectories.

(a) Two cubic-order trajectories;						(b) Two quadratic-order trajectories					
Model with two trajectories with order 3 3						Model with two trajectories with order 2 2					
Group	Parameter	Estimate	Standard error	*T* for *H*_0_: parameter = 0	Prob > |*T*|	Group	Parameter	Estimate	Standard error	*T* for *H*_0_: parameter = 0	Prob > |*T*|
1	Intercept	38.89033	2.93627	13.245	0.0000	1	Intercept	35.53250	1.30387	27.252	0.0000
	Linear	−0.00000	0.01905	−0.000	1.0000		Linear	−0.00974	0.00663	−1.469	0.1428
	Quadratic	−0.00000	0.00005	−0.058	0.9541		Quadratic	0.00001	0.00001	1.843	0.0662
	Cubic	0.00000	0.00000	0.112	0.9111						
2	Intercept	52.96765	2.30089	23.021	0.0000	2	Intercept	51.05711	1.02509	49.807	0.0000
	Linear	−0.00000	0.01658	−0.000	1.0000		Linear	0.00179	0.00426	0.419	0.6758
	Quadratic	−0.00000	0.00004	−0.086	0.9317		Quadratic	−0.00000	0.00000	−0.548	0.5841
	Cubic	0.00000	0.00000	0.070	0.9440						
	Sigma	10.55799	1.01720	10.379	0.0000		Sigma	7.41392	0.30445	24.352	0.0000
Group membership						Group membership					
1	(%)	50.00000	14.50867	3.446	0.0006	1	(%)	33.29470	5.33959	6.235	0.0000
2	(%)	50.00000	14.50867	3.446	0.0006	2	(%)	66.70530	5.33959	12.493	0.0000
BIC = −1257.43 (*N* = 332) BIC = −1251.86 (*N* = 109) AIC = −1238.41 ll = −1228.41						BIC = −1217.89 (*N* = 332) BIC = −1213.44 (*N* = 109) AIC = −1202.67 ll = −1194.67					
Entropy = 0.547						Entropy = 0.856					

Group: number of tested trajectories.

Parameter: shape of the trajectories.

Estimate and standard error: estimated values and standard error of parameters

*T* for *H*_0_: Parameter = 0: a value for the test of the null hypothesis that determines whether the parameter is significant or not

Prob > |*T*|: significance for each parameter (*p*-value).

Sigma: the amount of variance in the data accounted for by the model and its significance is given by Sigma. Group membership: estimated percentage of subjects belonging to each trajectory with standard error test and significance.

Two BIC values are provided in each output (the first is related to all observations, *n* = 332; the second is related to subjects, *n* = 109), the second BIC value has to be interpreted as an index of model fitting.

ll: log-likelihood

Entropy: values of entropy of model.

**Table 3:** Stata outputs of model with three trajectories.

(a) Three cubic-order trajectories;						(b) Three quadratic-order trajectories					
Model three trajectories with order 3 3 3						Model three trajectories with order 2 2 2					
Group	Parameter	Estimate	Standard error	*T* for *H*_0_: parameter = 0	Prob > |*T*|	Group	Parameter	Estimate	Standard error	*T* for *H*_0_: parameter = 0	Prob > |*T*|
1	Intercept	35.37100	2.35760	15.003	0.0000	1	Intercept	35.37460	1.35144	26.175	0.0000
	Linear	0.00000	0.02428	0.000	1.0000		Linear	−0.01781	0.00701	−2.541	0.0115
	Quadratic	−0.00001	0.00006	−0.193	0.8472		Quadratic	0.00002	0.00001	2.518	0.0123
	Cubic	0.00000	0.00000	0.337	0.7364						
2	Intercept	45.92899	1.87334	24.517	0.0000	2	Intercept	37.93594	2.16515	17.521	0.0000
	Linear	0.00001	0.02199	0.000	0.9998		Linear	0.02756	0.00914	3.016	0.0028
	Quadratic	0.00002	0.00005	0.354	0.7238		Quadratic	−0.00002	0.00001	−2.406	0.0167
	Cubic	−0.00000	0.00000	−0.481	0.6309						
3	Intercept	56.48698	1.37030	41.222	0.0000	3	Intercept	54.10621	1.07422	50.368	0.0000
	Linear	−0.00001	0.02014	−0000	0.9998		Linear	−0.00595	0.00451	−1.319	0.1881
	Quadratic	−0.00002	0.00005	−0.501	0.6166		Quadratic	0.00000	0.00000	0.759	0.4484
	Cubic	0.00000	0.00000	0.634	0.5267						
	Sigma	10.55773	0.76489	13.803	0.0000		Sigma	6.55832	0.28621	22.915	0.0000
Group membership						Group membership					
1	(%)	33.33333	8.08421	4.123	0.0000	1	(%)	25.60246	4.98020	5.141	0.0000
2	(%)	33.33334	7.69248	4.333	0.0000	2	(%)	21.29235	6.23463	3.415	0.0007
3	(%)	33.33333	9.51886	3.502	0.0005	3	(%)	53.10519	6.24921	8.498	0.0000
BIC = −1270.70 (*N* = 332) BIC = −1262.35 (*N* = 109) AIC = −1242.16 ll = −1227.16						BIC = −1217.07 (*N* = 332) BIC = −1210.39 (*N* = 109) AIC = −1194.24 ll = −1182.24					
Entropy = 0.409						Entropy = 0.801					

### Step 4–assess the model fit

We then examined models with four trajectories of cubic and quadratic order (see Extra Table S1). Given the fact that the BIC values were higher for models with four trajectories (cubic order: −1275.54 and quadratic order: −1217.17) as compared to those reported in Table 3 based on three trajectories (cubic order: −1262.35 and quadratic order: −1210.39), we focused on the three-trajectories quadratic model because it best fits the data. To identify the pattern of individual group trajectories, it is necessary to analyze the statistical significance of the estimated coefficients (intercept, linear, quadratic, or cubic; in the output of Stata software, the significance of a single variable is indicated with Prob > |*T*|). Examining the *p*-values associated with the various model orders in Table 3b, the quadratic order was suitable for the first two trajectories (*p* = 0.012 and *p* = 0.017) but not for the 3rd trajectories (*p* = 0.45). Thus, for the final trajectories, we decreased the order from quadratic to linear (Table 4a) and intercept (Table 4b).

**Table 4:** Model Stata outputs with three trajectories adopting different orders (“2 2 0” and “2 2 1”).

(a) Three trajectories (quadratic order, *n* = 2; only intercept, *n* = 1)						(b) Three trajectories (quadratic order, *n* = 2; linear order, *n* = 1)					
Model three trajectories with order 2 2 0						Model three trajectories with order 2 2 1					
Group	Parameter	Estimate	Standard error	*T* for *H*_0_: parameter = 0	Prob > |*T*|	Group	Parameter	Estimate	Standard error	*T* for *H*_0_: parameter = 0	Prob > |*T*|
1	Intercept	35.41126	1.39221	25.435	0.0000	1	Intercept	35.38416	1.35655	26.084	0.0000
	Linear	−0.01799	0.00711	−2.53	0.0119		Linear	−0.01784	0.00703	−2.539	0.0116
	Quadratic	0.00002	0.00001	2.501	0.0129		Quadratic	0.00002	0.00001	2.512	0.0125
2	Intercept	37.54629	2.46310	15.244	0.0000	2	Intercept	37.81315	2.19586	17.220	0.0000
	Linear	0.02678	0.01046	2.559	0.0109		Linear	0.02691	0.00922	2.920	0.0037
	Quadratic	−0.00002	0.00001	−2.066	0.0396		Quadratic	−0.00002	0.00001	−2.300	0.0221
3	Intercept	52.34803	0.63467	82.481	0.0000	3	Intercept	53.70653	0.92624	57.984	0.0000
							Linear	−0.00269	0.00135	−1.994	0.0470
	Sigma	6.66619	0.29185	22.841	0.0000		Sigma	6.57488	0.28672	22.931	0.0000
Group membership						Group membership					
1	(%)	25.54122	5.05856	5.049	0.0000	1	(%)	25.57552	4.97457	5.141	0.0000
2	(%)	19.36005	6.43961	3.006	0.0028	2	(%)	20.77718	6.16119	3.372	0.0008
3	(%)	55.09873	6.27593	8.779	0.0000	3	(%)	53.6473	6.17406	8.689	0.0000
BIC = −1213.62 (*N* = 332) BIC = −1208.05 (*N* = 109) AIC = −1194.59 ll = −1184.59						BIC = −1214.47 (*N* = 332) BIC = −1208.34 (*N* = 109) AIC = −1193.54 ll = −1182.54					
Entropy = 0.793						Entropy = 0.801					

**Figure 1:**
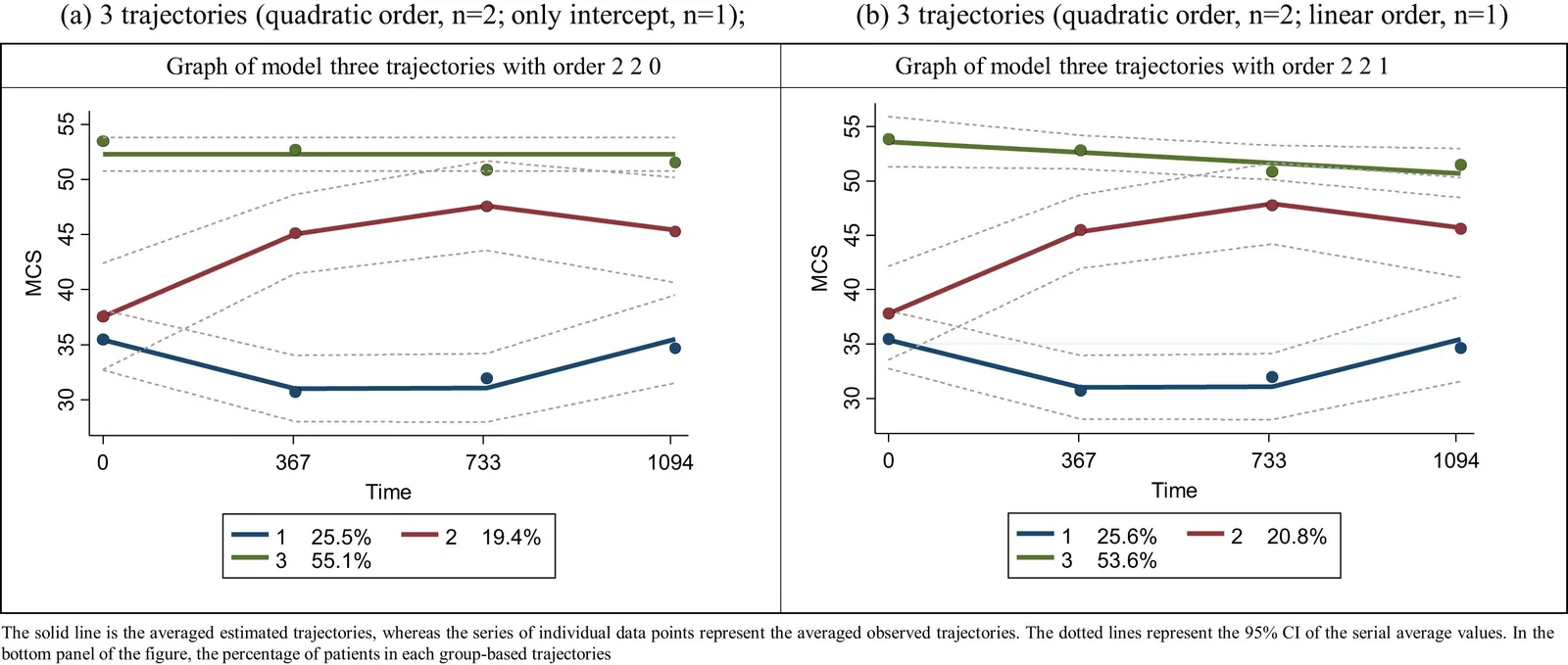
Trajectories of MCS evolution over time adopting (a) “2 2 0” and (b) “2 2 1” order.

Based on the *p*-value analysis (*p* < 0.0001) and BIC (−1208.05), the model having three trajectories with orders “2 2 0” seems to be more adequate than that with three trajectories with orders of “2 2 1” (*p* = 0.047 and BIC −1208.34). However, by comparing observed and predicted values (see Fig. 1), it is evident that estimated values are closer to those observed when adopting three trajectories with order “2 2 1” (Fig. 1b) instead of three trajectories with order 2 2 0 (Fig. 1a). This emphasizes the importance of combining the statistical model results with the visual inspection of figures plotting observed and predicted values. Notably, the resulting groups include more than 5% of the total cases (Fig. 1 and Table 5).

**Table 5:** MCS score—BIC according to the number of groups, trajectories shapes and entropy.

Number of groups	Trajectories patterns	BIC (*N* = 332)	BIC (*N* = 109)	Entropy	Groups with <5% of patients
2	3 3	−1257.43	−1251.86	0.547	0
2	2 2	−1217.89	−1213.44	0.856	0
3	3 3 3	−1270.70	−1262.35	0.408	0
3	2 2 2	−1217.07	−1210.39	0.801	0
4	3 3 3 3	−1286.68	−1275.54	0.337	0
4	2 2 2 2	−1226.08	−1217.17	0.751	0
3	2 2 0	−1213.62	−1208.05	0.793	0
3	2 2 1	−1214.47	−1208.34	0.801	0

By adopting the model “2 2 1”, the percentages of patients in each group were 25.6%, 20.8%, and 53.6% (see Fig. 1b).

Overall, the MCS variable follows three distinct paths over time (see Fig. 1b). The first is a linear decline (see upper trajectory in Fig. 1b), where the value steadily decreases at a constant rate, showing a clear and continuous downward trend. By contrast, the third trajectory presents a quadratic pattern: the variable initially drops but then begins to rise slightly, suggesting a recovery after an initial decline (see bottom trajectory in Fig. 1b). The second trajectory also follows a quadratic trend, though in a different direction—the variable increases during the early stages, but this growth gradually slows down and eventually levels off, resulting in a plateau (see intermediate trajectory in Fig. 1b).

Furthermore, in order to select the best fit model, it is also necessary to take into account:

- the APP of group membership, which must be greater than 0.70 in each trajectory; this suggests that the modeled trajectories categorize individuals who exhibit similar trends in their changes and discriminate between individuals exhibiting different patterns of change over the considered time. (The APPs associated with the trajectories “2 2 1” were 95.0%, 81.0%, and 93.0%, respectively);
- the Odds of correct classification must be >5 (OCC associated with the trajectories “2 2 1” were 60.4, 16.8, and 12.4, respectively);
- entropy, which varies between 0.00 and 1.00, is considered as a summary indicator of the conditional probabilities related to individuals’ group membership: high values (greater than 0.80) suggest that individuals are accurately classified and that there is a clear distinction between the latent classes. The entropy was also adequate (0.801).

Given that all criteria are met, the best-fitting model includes three trajectories: two quadratic and one linear. This classification can be used to examine the demographic and clinical characteristics of patients in each group, as well as the relationship between this categorization and future clinical outcomes.

## CONCLUSIONS

Trajectory analysis is a method that provides nuanced insights into patterns of change in patient outcomes over time. By applying advanced statistical approaches, researchers can uncover distinct subgroups and trends that traditional methods might miss.

Although GBTM yields latent statistical classes should not be interpreted a priori as definitive biological entities, the identified trajectory patterns may still represent meaningful clinical phenotypes [7]. In nephrology, these patterns have been linked to varying risks of cardiovascular events, ESKD (End-Stage Kidney Disease) progression, and mortality, highlighting their relevance beyond a purely statistical framework. Importantly, membership in a given trajectory group characterizes patients with distinct risk profiles for clinically relevant outcomes, thereby reinforcing the prognostic value of these longitudinal patterns [13].

For practical reasons due to restriction related to data access and ownership, we used a hypothetical cohort. As a consequence, data presented in this paper may not capture all the complexities of real-world settings.

In the current example, the analysis focuses on the description of different trajectories, resulting in the identification of three distinct groups of patients. A logical next step would be to compare clinical outcomes across these groups, as has been demonstrated in other studies (e.g. the MAURO (Multiple Intervention and Audit in Renal Diseases to Optimize Care) study [7]). This approach, which classifies patients based on the evaluation of specific variables over time, allows the identification of phenotypic groups according to their baseline characteristics and enables investigation of the prognostic impact of this classification on future clinical outcomes, thereby supporting targeted interventions and personalized treatment strategies.

## Supplementary Material

sfag239_Supplemental_File

## Data Availability

No new data were generated or analyzed in support of this research.
